# Transcatheter Interventions for Atrioventricular Dysfunction in Patients with Adult Congenital Heart Disease: An International Case Series

**DOI:** 10.3390/jcm12020521

**Published:** 2023-01-09

**Authors:** Nili Schamroth Pravda, Hana Vaknin Assa, Lars Sondergaard, Vilhelmas Bajoras, Horst Sievert, Kerstin Piayda, Amos Levi, Guy Witberg, Yaron Shapira, Ashraf Hamdan, Leor Perl, Shahar Vig, Leonard Blieden, Ran Kornowski, Rafael Hirsch, Pablo Codner

**Affiliations:** 1 Department of Cardiology, Rabin Medical Center, Petah Tikva 4941492, Israel; 2 Faculty of Medicine, Tel Aviv University, Tel Aviv 6997801, Israel; 3 Department of Cardiology, Rigshospitalet, University of Copenhagen, 2100 Copenhagen, Denmark; 4 CardioVascular Center Frankfurt CVC, 60389 Frankfurt am Main, Germany

**Keywords:** ACHD, atrioventricular valve, transcatheter

## Abstract

Introduction: A substantial proportion of patients with adult congenital heart disease (ACHD) suffer from worsening valvular dysfunction in adulthood. Transcatheter valve interventions can offer a therapeutic alternative to surgery for those at high surgical risk. There is emerging but limited data on transcatheter interventions for atrioventricular (AV) valve dysfunction in patients with ACHD. Methods: We compiled an international collaborative multi-center registry focusing on adult patients with congenital heart disease undergoing transcatheter AV valve interventions (repair or replacement). Included were patients from three international centers who underwent procedures between 2016 and 2022. Demographic, clinical, and procedural data were compiled. Results: Nine patients with ACHD underwent AV valve interventions. The median age was 48 years (IQR (37; 56), 55% women). At baseline, seven patients (78%) were in NYHA functional class III and two (22%) were in NYHA functional class II. The diagnosis of ACHD varied. Three valve interventions were performed on the subpulmonary AV valve and six on the systemic AV valve. The primary valvular pathology was regurgitation (six patients, 78%). Five procedures were valve-in-valve interventions, and four procedures were transcatheter edge-to-edge repair procedures. There were no major complications or peri-procedural complications or peri-procedural mortality. One patient developed a suspected non-obstructive thrombus on the valve that was medically treated. One patient did not improve clinically following the procedure and underwent a heart transplant, one patient died 6 months following the procedure due to a cardiovascular implantable electronic device infection. At one year, six patients were in NYHA functional class I, and one patient was in NYHA functional class III. In conclusion, transcatheter AV heart valve interventions are feasible and safe procedures in carefully selected ACHD patients. These procedures can offer an effective treatment option in these younger patients with high surgical risk.

## 1. Introduction

The prevalence of patients with congenital heart disease surviving into adulthood is increasing due to medical and surgical advances [1,2]. A significant portion of these patients suffer from valvular dysfunction with ensuing morbidity and mortality [3]. Many of these patients have undergone multiple prior surgical interventions in childhood and have enhanced risk factors for structural valve deterioration when advancing into adulthood and older age. This is more pertinent with the demographics of the ACHD population having increased longevity [4]. Transcatheter valve intervention offers a therapeutic alternative to those at high surgical risk [5]. There are validated data on the use of transcatheter interventions in the pulmonary valve position and substantial observational data on transcatheter aortic valve interventions, specifically in those with bicuspid aortic valve associated pathology [6]. However, there are sparse data on transcatheter intervention in the AV valves in patients with adult congenital heart disease (ACHD).

## 2. Methods

We compiled an international collaborative multi-center registry focusing on ACHD patients undergoing transcatheter AV heart valve interventions (repair or replacement). Included were patients from 3 international centers who underwent procedures between 2016 and 2022. Demographic, clinical, and procedural data were compiled. All patients were assessed in the setting of a local heart team and were favored for transcatheter management taking into consideration multiple parameters including anatomical complexity of the procedure, re-operative risk, etc.

### Statistical Analysis

Descriptive statistics were used to tabulate and summarize baseline, procedural, and follow-up data. Categorical data are presented as numbers and percentages of the total; continuous variables are shown as means with standard deviations or medians with 25th and 75th percentiles (Q1, Q3) for non-normally distributed data. This project received ethics board approval.

## 3. Results

Included were nine patients with ACHD who underwent AV valve interventions. The median age was 48 years (IQR (37; 56), 55% women). At baseline, seven patients (78%) were in NYHA functional class III, and two (22%) were in NYHA functional class II. The diagnosis of ACHD varied, as shown in Table 1. Three valve interventions were performed on the subpulmonary AV valve and six on the systemic AV valve. The primary valvular pathology was regurgitation (six patients, 78%). Five procedures were valve-in-valve interventions, and four procedures were transcatheter edge-to-edge repair procedures. One patient developed a suspected non-obstructive thrombus on the valve that was medically treated. One patient had a vascular complication following the procedure of femoral pseudo-aneurysm, and one patient developed an atrial flutter acutely following the procedure. There were one-year follow-up data for seven patients. At one year, six patients were in NYHA functional class I, and one patient was in NYHA functional class III. One patient did not clinically improve following the procedure and underwent a heart transplant, and one patient died during follow up due to a cardiovascular implantable electronic device infection. A detailed description of each case is found below and summarized in Table 1.

### 3.1. Case 1

A female patient in her sixth decade of life had a past history of primary lesion of a discrete sub-aortic membrane that had been resected and an ensuing Ross–Konno procedure and tricuspid valve replacement in her fifth decade of life due to severe tricuspid regurgitation with implantation of an Epic 33 mm valve. She was sent for AV valve intervention due to tricuspid bioprosthesis failure with severe regurgitation and stenosis. The patient had significant clinical signs of peripheral congestion with ascites and evidence of liver congestion on laboratory findings and was clinically in NYHA class III. On echocardiogram evaluation, the peak tricuspid valve pressure was 18 mmHg with a mean of 13 mmHg. The inferior vena cava was enlarged to 35 mm and did not undergo respiratory variation. Left and right ventricular systolic function were normal with a left ventricular ejection fraction of 60% and TAPSE 22 mm. The case was discussed in the heart team forum, and the patient was referred for tricuspid valve-in-valve (VIV) intervention, which was performed under conscious sedation via femoral vein access. A Sapien 3 29 mm (Edwards, Irvine, CA, USA) valve was inserted with a reduction in the peak tricuspid gradient to 6 mmHg and a mean gradient of 4 mmHg, as shown in Video S1. There were no complications during follow up.

### 3.2. Case 2

**Figure 1:**
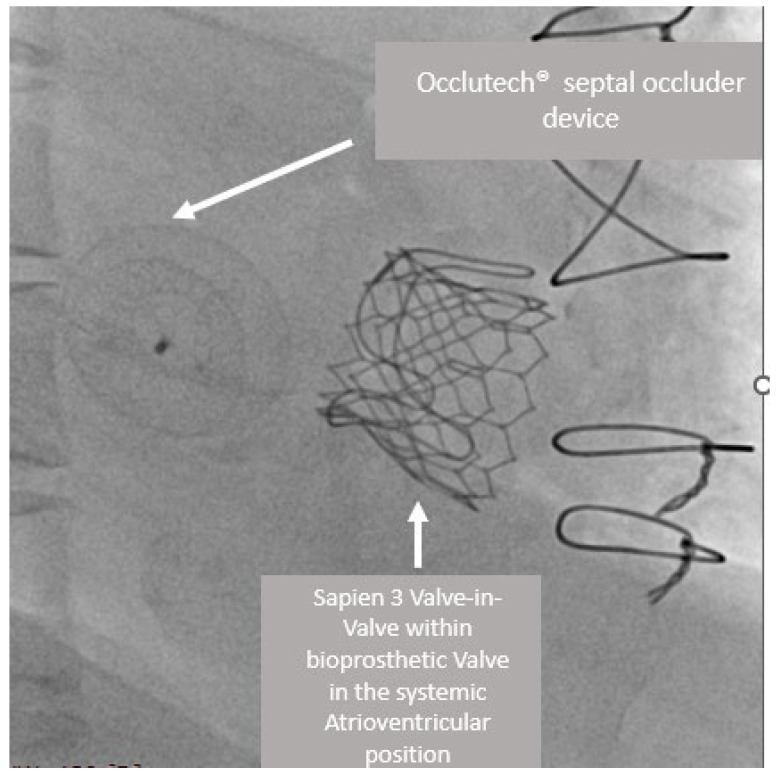
Sapien 3 valve-in-valve within bioprosthetic Valve in the systemic atrioventricular position with Occlutech^®^ septal occluder device as shown in the RAO view.

A female patient with congenitally corrected transposition of the great arteries had previously undergone replacement of the systemic AV valve with a Carpentier Edwards 25 mm bioprosthesis. She presented in the fourth decade of life with acute decompensated heart failure with severe structural deterioration of the bioprosthesis with mixed pathology of regurgitation and stenosis. On echocardiographic examination, the systemic ventricle had preserved systolic function, and the bioprosthetic valve had severe stenosis with a gradient of peak 45 mmHg with a mean of 20 mmHg as well as a flail leaflet with severe regurgitation. A Sapien 3 26 mm valve was inserted via femoral vein/transseptal access with hemodynamic improvement and residual minimal regurgitation and prosthetic patient mismatch with mild–moderate stenosis with an 8 mmHg mean pressure gradient. Due to a significant left-to-right shunt via the iatrogenic atrial septal defect and subsequent desaturation, an Occlutech^®^ (Occlutech GmbH, Jena, Germany) septal occluder device 13.5 mm was inserted at the end of the procedure. This can be seen in Figure 1. The patient had a subsequent episode of atrial flutter and bradycardia, which was medically managed. She has not had a subsequent admission for heart failure and has been clinically well.

### 3.3. Case 3

**Figure 2:**
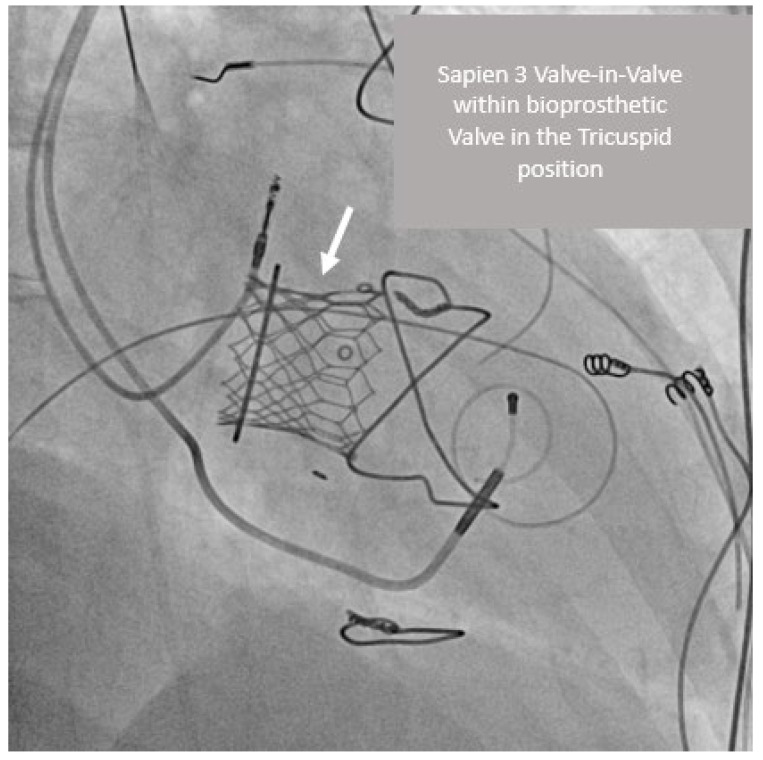
Sapien 3 valve-in-valve within bioprosthetic valve in the tricuspid position with pacemaker leads in situ in the RAO38 Caudal 10 view.

A female patient was known with Ebstein anomaly, after mechanical tricuspid valve replacement in childhood and subsequent atrioventricular block with the need for a pacemaker. The patient previously had a non-obstructive stuck valve during pregnancy and had repeat surgery with the insertion of a 31 mm Hancock bioprosthesis 23 years prior to presentation. She presented in the fourth decade of life with valve structural deterioration with mixed moderate regurgitation, and stenosis with a mean gradient of 10 mmHg. The patient was referred for VIV intervention. The procedure was performed via femoral vein access with implantation of a Sapien 3 29 mm valve with no residual regurgitation and mean gradient of 2 mmHg. This is shown in Figure 2 and Video S2. Following the procedure, the patient has been clinically well—NYHA functional class I and no subsequent admissions.

### 3.4. Case 4

**Figure 3:**
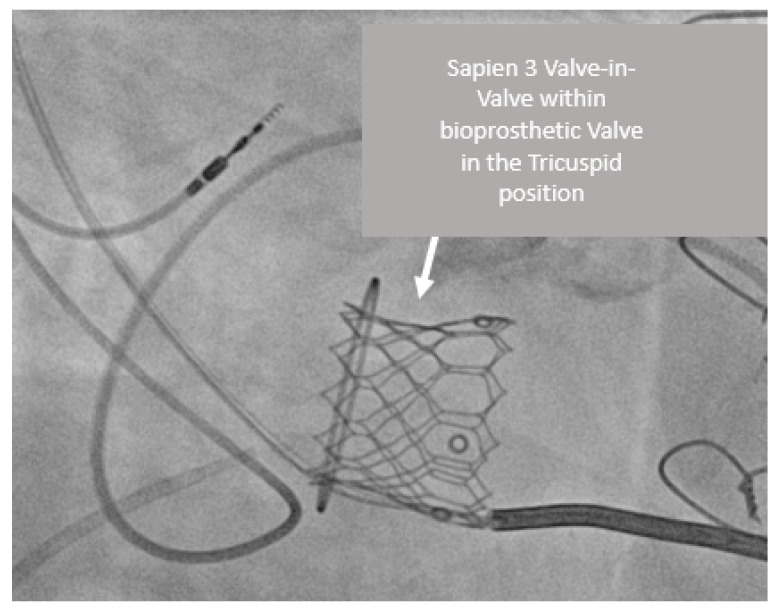
Sapien 3 valve-in-valve within bioprosthetic valve in the tricuspid position with cardiac resynchronization therapy defibrillator device in-situ in the RAO 50 Caudal 10 view.

A man in his sixth decade of life had a primary complex cyanotic cardiac defect of the double outlet right ventricle with pulmonary stenosis and underwent a Rastelli repair operation during his youth with the need for an epicardial pacemaker due to symptomatic bradycardia. He previously had a surgery in which a pulmonary homograft was implanted, the tricuspid valve was repaired, and a residual ventricular septal defect was closed. In the following years, the patient suffered from episodes of ventricular tachycardia, and the functioning of the tricuspid valve deteriorated and was replaced with a Hancock 33 mm bioprosthesis. The tricuspid underwent structural valve deterioration with mixed pathology of stenosis and regurgitation and, due to the accelerated valvular deterioration, anticoagulation therapy was initiated. The patient suffered from right heart failure with severe hypoalbuminemia from protein-losing enteropathy. The tricuspid valve underwent structural deterioration with severe tricuspid regurgitation. The echocardiographic assessment showed severe tricuspid regurgitation with vena contracta of 8 mm, an AV valve peak gradient of 8 mmHg, and a mean of 6 mmHg and an estimated systolic pulmonary artery pressure of 73 mmHg. The patient was referred for a tricuspid valve-in-a valve procedure. The procedure was performed via femoral vein access, and a Sapien 3 valve of 29 mm was inserted into the tricuspid bioprosthesis (Figure 3). The AV valve peak gradient dropped to 7 mmHg with a mean of 5 mmHg, with no residual tricuspid regurgitation. A hemodynamic invasive assessment following the procedure showed right ventricular end diastolic pressure of 9 mmHg, normal pulmonary artery pressures, and right AV valve mean pressure gradient of 7 mmHg, most likely due to an element of calcified tricuspid stenosis. Five months later, the patient developed a cardiac device infection and had a complicated admission, and ultimately died.

### 3.5. Case 5

This patient was in his second decade of life with idiopathic dilated cardiomyopathy from a young age with severe left ventricular dysfunction. He had significant heart failure, and his baseline NYHA function class was grade III and a known ejection fraction <30%. He had severe secondary mitral regurgitation with peak pulmonary artery pressure measured as 75 mmHg. A transcatheter edge-to-edge repair of the mitral valve was performed using a Mitraclip^TM^ device. This reduced the mitral regurgitation to mild with moderate pulmonary hypertension. However, the patient remained clinically in NYHA functional class III and, one year later, underwent a heart transplant.

### 3.6. Case 6

A female patient in her eighth decade of life had cor triatriatum sinister Löffler III and concomitant severe mitral regurgitation. She was in symptomatic heart failure in NYHA functional class III.

On echocardiography, she had a preserved ejection fraction with severe mitral regurgitation. She was referred for transcatheter edge-to-edge mitral valve repair with a Mitraclip™ device. On echocardiography following the procedure, the mitral regurgitation was reduced to mild with mild mitral stenosis and an increase in systolic pulmonary artery pressure from 17 to 37 mmHg.

### 3.7. Case 7

A man in seventh decade of life with known atrial septal defect (ASD) with total pulmonary vein confluence previously underwent surgery with insertion of an ASD patch and implantation of a 28 mm Starflex-Occluder due to a residual shunt. He presented with severe mitral regurgitation and underwent transcatheter indirect mitral valve banding with Carillon^®^ Mitral Contour System® (Cardiac Dimensions, Kirkland, WA, USA) 12–20–60 mm. A vascular complication of a femoral pseudoaneurysm occurred during the procedure that was treated with thrombin injection. Following the procedure, the mitral regurgitation was reduced to moderate with no mitral stenosis and systolic pulmonary artery pressure decreased from 58 to 31 mmHg.

### 3.8. Case 8

A male patient in his fourth decade of life was born with D transposition of the great arteries and underwent Senning atrial switch operation as a child. The patient developed moderate-to-severe systemic AV regurgitation with mildly reduced function of the systemic (right) ventricle. He remained clinically stable over the years and underwent cardiac MRI that showed moderate tricuspid regurgitation with an ejection fraction of the systemic ventricle of 41% and an end diastolic volume of 166 mL/m^2^. The patient contracted CCOVID-19, which worsened heart failure symptoms. He was stabilized on heart failure medication and subsequently underwent a cardiac MRI, which showed a reduced EF of 35%, end diastolic volume of the systemic (right ventricle) of 198 mL/m^2^, and moderate-to-severe systemic AV valve regurgitation with regurgitant fraction of 38%. The patient remained in NYHA functional class III despite optimal medication therapy and was referred for the Mitraclip ™ procedure. The procedure was conducted via femoral vein access with puncture from the pulmonary to systemic venous baffle. Under TEE guidance, the tricuspid valve was seen with two leaflets only (anterior and posterior). Two Mitraclip™ XT clips were placed between the anterior and posterior leaflets, with assistance of the Triclip™ systemic for flexibility. At the end of the procedure, an iatrogenic atrial septal defect of 5 by 7 mm was found, and this was closed by an Amplatzer Flex II 10.5 mm occluder device. The systemic AV regurgitation was reduced to minimal with a mean AV valve gradient of 4 mmHg. One month following the procedure, the patient presented with heart failure and atrial flutter. This improved following cardioversion to sinus rhythm.

### 3.9. Case 9

A female patient in her fifth decade of life had congenitally corrected transposition of the great vessels. She had undergone systemic valve replacement with a mechanical valve in her youth, which was replaced with a biological valve (Hancock 25 mm) following valve thrombosis. The patient also had a pacemaker inserted due to complete atrioventricular block in her youth, and this had been replaced by a cardiac resynchronization device with an epicardial lead. She had been medically managed for atrial arrythmias. The patient developed symptoms of decompensated heart failure and underwent evaluation. On transesophageal echocardiography, the regurgitation was intra-valvular and graded as moderate to severe with a pressure gradient of peak 22 mmHg and a mean gradient of 6 mmHg over the bioprosthesis in the presence of severe systolic systemic ventricular dysfunction. A hemodynamic catheterization demonstrated postcapillary pulmonary hypertension with mixed valvular dysfunction (mean wedge pressure of 18 mmHg with left atrial V waves of 22 mmHg with a reduced cardiac index of 1.9 L/minute/m^2^, pulmonary hypertension with PVR 8.6 Woods, and mitral valve area calculated to 1.3 cm^2^). Following a heart team discussion, it was decided that the mixed valvular dysfunction was significant, and a valve-in-valve procedure was performed under general anesthesia with a trans-septal approach with the implantation of a Sapien S3 26 mm valve. Balloon dilation was performed following the implantation. The TEE assessment following the implantation showed that the valve gradients were reduced to a peak of 9 mmHg and a mean of 5 mmHg. During the admission following the procedure, there was a concern that there was non-obstructive thrombus on the valve, and oral anticoagulation was changed to warfarin therapy. Following the procedure, the ventricular function did not improve. However, at the 6-month follow up, the patient reported a subjective improvement and was clinically stable on heart failure medications.

## 4. Discussion

In this case series, we described nine patients with ACHD that underwent AV valve interventions. The ACHD diagnoses were varied, and the primary AV valve pathology was predominantly that of regurgitation. Five procedures were valve-in-valve interventions, and four procedures were transcatheter edge-to-edge repair procedures. The procedures provided hemodynamic and clinical improvement in most patients.

This collaborative registry provides insights into patients with complex anatomy needing innovative interventions. Patients with congenital heart disease are surviving into adulthood due to improved surgical and interventional techniques [2]. With these patients surviving into older age, there is an increasing incidence of valvular dysfunction [3]. Our results are optimistic in showing that percutaneous valvular interventions are a feasible treatment option in this patient population—patients who are often at high surgical risk and who have had multiple prior cardiac interventions/surgeries. There have been enormous advances in the field of transcatheter valvular interventions such as aortic valve implantation and mitral valve repair. Additionally, international guidelines support the use of these therapies, especially in those with high surgical risk [7,8]. In the field of ACHD, there is increasing evidence to support the use of transcatheter valve insertion in the semilunar valves as an alternative to surgical intervention in those at high surgical risk—both in the aortic position in patients with bicuspid aortic valve and for patients with pulmonary valve disease [8,9]. However, there are sparse data on the use of AV valve transcatheter interventions in the ACHD population. Alshawabkeh et al. published a case series of three patients with congenital heart disease who successfully underwent percutaneous mitral valve repair [10]. Prior to this, only case reports had been published. Silini et al. compiled a review of the case report and series published in the literature including that of Alshawabkeh et al. [11] While each case series was small in number, the data on these procedures are slowly increasing in the literature. Silini et al. concluded that while the procedures seem safe and yield clinical improvements, the specific complex anatomy of each case must be carefully assessed and that long-term outcome data are lacking [11]. The data from our case series are the largest reported and add to limited but ever-increasing data on these complex interventions in this unique patient population [11,12]. Ott et al. reported on six patients with congenitally corrected transposition of the great arteries who underwent transcatheter edge-to-edge repair with Mitraclip in the systemic tricuspid valve with favorable clinical results at one-year follow up. This case series highlights the importance of knowing the underlying anatomical challenges prior to intervention and the value of ingenuity when using interventions such as Mitraclip on the structural tricuspid valve in the systemic position [12].

Transcatheter tricuspid valve-in-valve has been shown to be an attractive treatment option in patients with Ebstein anomaly and a failing tricuspid bioprosthesis. Taggart et al. reported on 81 patients who underwent transcatheter tricuspid valve-in-valve implantation with an average follow up of 13 months. The majority had an improvement in functional class, and valve hemodynamics was improved. On subgroup analysis, they found that patients 19 to 40 years old had significantly shorter freedom from reintervention or valve dysfunction with an odds ratio of 8.02 (*p* = 0.001) [13]. This highlights the need for accumulating data on these interventions to assess for long-term durability. Many ACHD patients present at a younger age than those with other valvular diseases; when deciding on surgical or transcatheter interventions, durability must be taken into consideration. Furthermore, many of these cases have complex anatomy and have unique challenges such as trans-baffle puncture in the cases of patients with transposition of the great arteries who have undergone an atrial switch procedure.

Our case series details a heterogenous patient population. ACHD is an umbrella term for multiple different pathologies. As described in our case series, each patient had different anatomical complexities that had to be treated innovatively. These cases were treated at tertiary centers with multidisciplinary teams with expertise in ACHD, imaging, and interventional cardiology. All cases were discussed with a heart team prior to intervention.

Our data have some important limitations. The main limitation is that of the small number of patients in this case series of unique patients. Each patient had different pathology and valve disease. Our results cannot be generalized to the broader ACHD population. Data regarding the nature of the initial congenital diagnosis and prior interventions from childhood were limited or lacking in most cases.

In conclusion, trans-catheter AV heart valve interventions are feasible and safe procedures in carefully selected ACHD patients. These procedures can offer an effective treatment option in these younger patients with high surgical risk and complex valvular lesions.

## Figures and Tables

**Table 1:** Summary of clinical and procedural characteristics of the cases.

Decade at Time of Intervention	Sex	ACHD Diagnosis	Number or Previous Sternotomies	Years Since Previous Valve Intervention	Systemic or Pulmonary AV Valve	Primary Valvular Pathology	AV Intervention	Follow-Up Complications
6th	Female	Discrete subaortic membrane. Previous Ross–Konno operation. Tricuspid bioprosthesis with SVD	3	7	Pulmonary	Regurgitation	VIV	None
4th	Female	CCTGA with systemic AV bioprosthesis SVD	2	9	Systemic	Stenosis	VIV	Atrial flutter/bradycardia
5th	Female	Ebstein anomaly with 2 previous tricuspid valve replacements and bioprosthesis SVD	2	23	Pulmonary	Mixed	VIV	None
6th	Male	Double outlet Right ventricle with pulmonary stenosis and residual VSD	4	9	Pulmonary	Mixed	VIV	Preserved valve function. Cardiovascular implantable electronic device (CIED) infection. Death.
Second	Male	Dilated cardiomyopathy with secondary mitral regurgitation	0	N/A	Systemic	Regurgitation	Transcatheter edge-to-edge repair with Mitraclip^TM^	Heart transplant
8th	Female	Cor triatriatum sinister Löffler III	0	0	Systemic	Regurgitation	Transcatheter edge-to-edge repair with Mitraclip^TM^	None
7th	Male	Persistent ASD with total pulmonary vein confluence following prior closure	1	1	Systemic	Regurgitation	Transcatheter edge-to-edge repair—Carillon device^TM^	Femoral pseudoaneurysm
4th	Male	D-TGA—senning atrial switch	1	33	Systemic	Regurgitation	Transcatheter edge-to-edge repair	Atrial flutter and tachycardia-induced dysfunction
5th	Female	CCTGA	2	9	Systemic	Regurgitation	VIV	Non-obstructive valve thrombus, minor vascular complication

SVD = structural valve deterioration, CCTGA = congenitally corrected transposition of the great arteries, AV = atrio-ventricular, VSD = ventricular septal defect, ASD = atrial septal defect, D-TGA = dextro-transposition of the great arteries, N/A = not applicable, VIV = valve-in-valve procedure.

## Data Availability

The data presented in this study are available on request from the corresponding author.

## Supplementary Materials

The following supporting information can be downloaded at: https://www.mdpi.com/article/10.3390/jcm12020521/s1, Video S1: Tricuspid Valve in Valve Procedure from case 1; Video S2: Tricuspid valve in valve in a patient born with Ebstein Anomoly.

## References

[1] Moons P. (2010). Patient-reported outcomes in congenital cardiac disease: Are they as good as you think they are?. Cardiol. Young.

[2] Moons P., Bovijn L., Budts W., Belmans A., Gewillig M. (2010). Temporal trends in survival to adulthood among patients born with congenital heart disease from 1970 to 1992 in Belgium. Circulation.

[3] Saef J.M., Ghobrial J. (2021). Valvular heart disease in congenital heart disease: A narrative review. Cardiovasc. Diagn. Ther..

[4] Moons P., Marelli A. (2022). Born to age: When adult congenital heart disease converges with geroscience. JACC Adv..

[5] Vahanian A., Beyersdorf F., Praz F., Milojevic M., Baldus S., Bauersachs J., Capodanno D., Conradi L., De Bonis M., De Paulis R. (2022). 2021 ESC/EACTS Guidelines for the management of valvular heart disease. Eur. Heart J..

[6] Vincent F., Ternacle J., Denimal T., Shen M., Redfors B., Delhaye C., Simonato M., Debry N., Verdier B., Shahim B. (2021). Transcatheter aortic valve replacement in bicuspid aortic valve stenosis. Circulation.

[7] Stout K.K., Daniels C.J., Aboulhosn J.A., Bozkurt B., Broberg C.S., Colman J.M., Crumb S.R., Dearani J.A., Fuller S., Gurvitz M. (2019). 2018 AHA/ACC guideline for the management of adults with congenital heart disease: A report of the american college of cardiology/american heart association task force on clinical practice guidelines. Circulation.

[8] Baumgartner H., De Backer J., Babu-Narayan S.V., Budts W., Chessa M., Diller G.-P., Lung B., Kluin J., Lang I.M., Meijboom F. (2021). 2020 ESC Guidelines for the management of adult congenital heart disease. Eur. Heart J..

[9] Hamdan A., Kornowski R. (2020). TAVI in bicuspid aortic valve stenosis. Int. J. Cardiol..

[10] Alshawabkeh L., Mahmud E., Reeves R. (2021). Percutaneous mitral valve repair in adults with congenital heart disease: Report of the first case-series. Catheter. Cardiovasc. Interv..

[11] Silini A., Iriart X. (2022). Percutaneous edge-to-edge repair in congenital heart disease: Preliminary results of a promising new technique. Int. J. Cardiol. Congenit. Heart Dis..

[12] Ott I., Rumpf P.M., Kasel M., Kastrati A., Kaemmerer H., Schunkert H., Ewert P., Tutarel O. (2021). Transcatheter valve repair in congenitally corrected transposition of the great arteries. EuroIntervention.

[13] Taggart N.W., Cabalka A.K., Eicken A., Aboulhosn J.A., Thomson J.D.R., Whisenant B., Bocks M.L., Schubert S., Jones T.K., Asnes J.D. (2018). Outcomes of Transcatheter Tricuspid Valve-in-Valve Implantation in Patients with Ebstein Anomaly. Am. J. Cardiol..

