# Supplementary figures and images for: Transcatheter Interventions for Atrioventricular Dysfunction in Patients with Adult Congenital Heart Disease: An International Case Series
Source: J Clin Med. 2023 Jan 9;12(2):521. doi: 10.3390/jcm12020521 (PMC9864755; doi:10.3390/jcm12020521)

## Slide 1
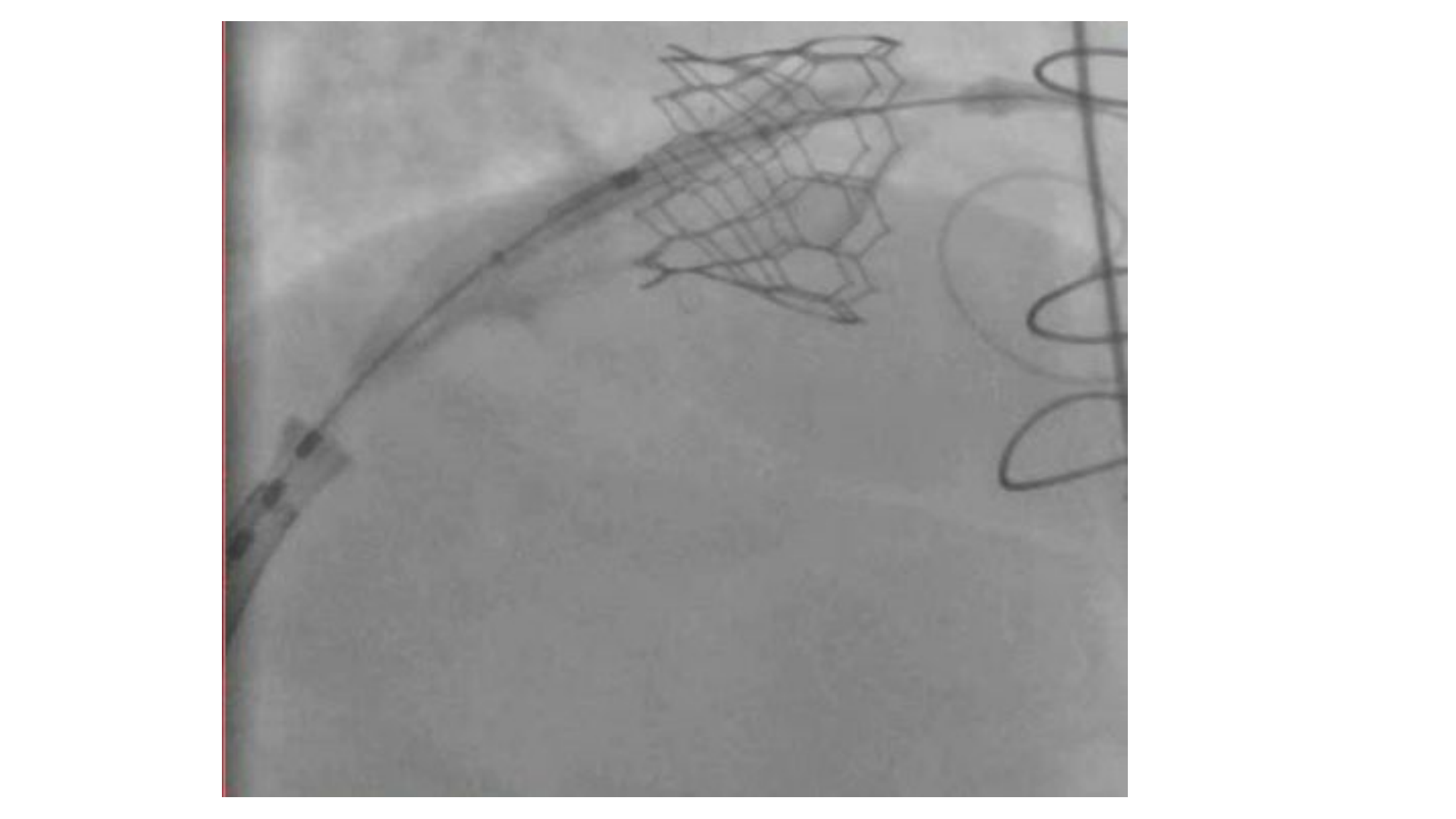

Supplement: Supplementary file 1 [file jcm-12-00521-s001.zip › Video S1.pptx]

## Slide 1
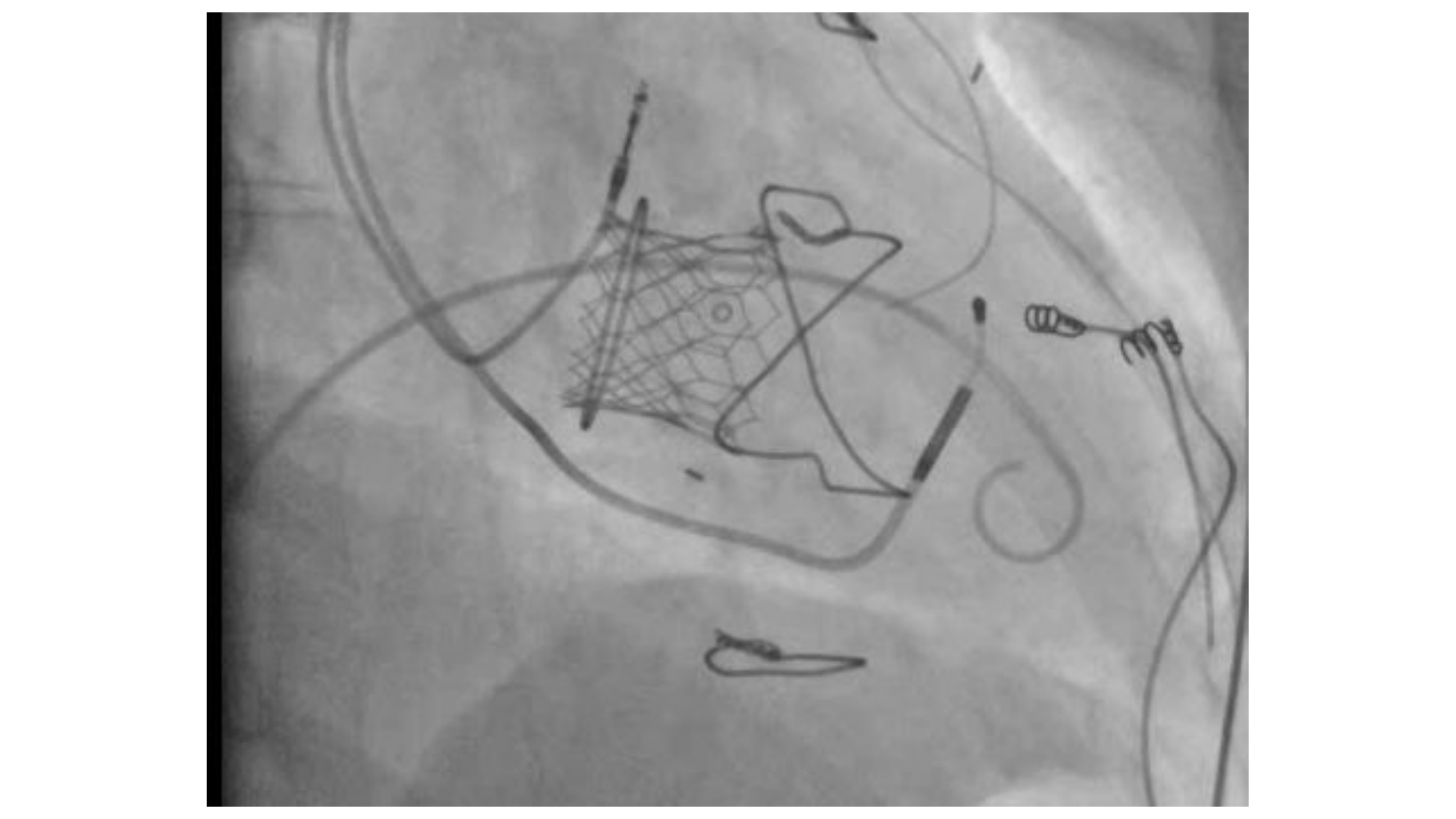

Supplement: Supplementary file 1 [file jcm-12-00521-s001.zip › Video S2.pptx]
